# Efficacy of a seal-wing paclitaxel-eluting balloon catheters in the treatment of bare metal stent restenosis

**DOI:** 10.1186/s12872-017-0602-6

**Published:** 2017-06-26

**Authors:** Leos Pleva, Pavel Kukla, Jana Zapletalova, Ota Hlinomaz

**Affiliations:** 1 Department of Cardiovascular Diseases, University Hospital Ostrava, tr. 17. listopadu 1790, 708 52 Ostrava, Czech Republic; 2 Department of Medical Biophysics, Palacky University, Hněvotínská 3, 775 15 Olomouc, Czech Republic; 3 Department of Cardioangiology, St. Anne’s University Hospital and Faculty of Medicine, Masaryk University, Pekařská 664/53, Brno, Czech Republic; 4 International Clinical Research Center, St. Anne’s University Hospital, Brno, Czech Republic

**Keywords:** In-stent restenosis, Paclitaxel-elution balloon, Drug-eluting stent

## Abstract

**Background:**

Our study aimed to compare the efficacy of seal-wing paclitaxel-eluting balloon catheters (PEB) with iopromide-coated PEB and everolimus-eluting stents (EES) for treating bare metal stent restenosis (BMS-ISR).

**Methods:**

We enrolled 64 patients with 69 BMS-ISR. The control group comprised patients from the iopromide-PEB and EES arms of a previous TIS study. The primary end-point was 12-month in-segment late lumen loss (LLL). Secondary end-points included incidence of binary in-stent restenosis and 12-month major adverse cardiac events (MACE).

**Results:**

Compared to iopromide-coated PEB, seal-wing PEB was associated with significantly higher 12-month LLL (0.30 vs. 0.02 mm; *p* < 0.0001), repeated binary restenosis (28.12% vs. 8.7%; *p* = 0.012), 12-month MACE (26.98% vs. 10.29%; *p* = 0.003), and target vessel revascularization (TVR; 20.63% vs. 7.35%; *p* = 0.009).

Compared to EES, no significant differences were found in the 12-month LLL (0.30 vs. 0.19 mm; *p* = 1.000), repeated binary restenosis (28.12% vs. 19.12%; *p* = 0.666), 12-month MACE (26.98% vs. 19.12%; *p* = 0.102) or TVR (20.63% vs. 16.18%; *p* = 0.360).

**Conclusion:**

BMS-ISR treatment using seal-wing PEB led to significantly higher 12-month LLL, repeated binary restenosis, MACE, and TVR compared to iopromide-coated PEB. However, no significant differences were found in comparison with EES.

**Trial registration:**

ClinicalTrials.gov; NCT01735825

## Introduction

Current treatments for in-stent restenosis utilize drug-eluting stents (DES) or drug-eluting balloon catheters (DEB) with locally released antiproliferative drugs. In contrast to DES, DEB allow short-term passage of the active substance (paclitaxel) into the vascular wall, preventing hyperproliferation of smooth muscle cells [1, 2]. Different paclitaxel-eluting balloon catheters (PEB) show varying efficacy, precluding discussion of “class effect” [3]. We demonstrated that in BMS-ISR treatment, iopromide-coated PEB resulted in significantly lower 12-month late lumen loss (LLL) compared to EES [4].

In our present study, we aimed to compare the effects of BMS-ISR treatment using PEB with different methods of paclitaxel binding to their surface.

## Methods

### Patients and study design

Our prospective, non-randomised study included consecutive adult patients (>18 years of age) with BMS-ISR (≥50% diameter stenosis; DS) who were treated with seal-wing PEB (Protége) in the Cathlab of University Hospital Ostrava in 2013–2015. The patients were followed 12 month after intervention and the study was finished in December 2016. The control group comprised patients with BMS-ISR who were treated using iopromide-coated PEB (Sequent Please) and EES (Promus; Pt/Cr metallic platform) in the previous randomized part of the TIS study [3]. The main exclusion criteria were concomitant diseases carrying expected survival times of <12 months or limiting the possibility of control coronary angiography (e.g., advanced renal failure), or inability to undergo long-term dual antiplatelet treatment (e.g., due to aspirin or clopidogrel allergy, bleeding complications, etc.).

The primary end-point was in-segment LLL at 12 months as measured by quantitative control angiography (QCA) [5]. Secondary end-points were the incidence of binary ISR (≥50% DS) and the overall incidence of 12-month major adverse cardiac events (MACE), including cardiovascular death, non-fatal acute myocardial infarction (MI), and target vessel revascularization (TVR).

### Interventions

PCI was performed under standard conditions from the radial or femoral approach, using a 6F guiding catheter and an Axiom X-ray system (Siemens AG, Forchheim, Germany).The patients were pre-treated with aspirin and clopidogrel (600 mg loading dose). Full anticoagulation was achieved by administering 100 IU/kg non-fractionated heparin, with a target activated clotting time of 250–300 s.

To pre-dilate the lesions and prevent any edge dissection, we used relatively shorter or scoring balloon catheters. Following predilatation, the PEB Protége (Blue Medical, Helmond, the Netherlands) was inflated for 30s at the recommended pressure. The seal-wing PEB Protége has paclitaxel (3 μg/mm^2^) tightly bound directly to the balloon catheter surface between the wings and hydrophilic coating prior to folding. This coating prevents releasing particles during bending of the balloon or transition to the stenosis. Paclitaxel, not coating, is only released when the balloon touches the vessel wall [6, 7].

Post-dilatation was performed using a non-compliant balloon catheter in the case of a suboptimal outcome, and another bailout stent was implanted in the case of edge dissection. All patients received standard therapy after coronary intervention. Dual antiplatelet therapy with aspirin (100 mg) and clopidogrel (75 mg) was administered daily for three months following PEB dilatation.

### Follow-up

Clinical follow-up was performed at 6 and 12 months. Angiographic follow-up was performed at 12 months (**±**2 months) unless needed earlier. All deaths were considered cardiac-related unless clearly from non-cardiac causes. Acute myocardial infarction was defined according to the third universal definition of myocardial infarction of the ESC [8], and stent thrombosis was defined based on ARC criteria [9].

### Angiographic follow-up

Prior to imaging, patients were administered intracoronary isosorbide dinitrate (1 mg). Imaging was performed using appropriate orthogonal projections to best avoid potential shortening or overlap of the reporting segment and lateral branches. Similar projections were used at the 12-month coronary angiography. Lesion type and ISR were evaluated in an in-segment section ±5 mm from the proximal and distal edges of the stent using ACC/AHA criteria [10] and Mehran’s classification [11]. Angiographic parameters were evaluated off-line using syngo Quantification software version 2007 (Siemens AG, Forchheim, Germany). The following parameters were measured: minimum lumen diameter (MLD), reference lumen diameter (RefD = ½ proximal + distal diameter), acute gain, lesion length, diameter of the stenosis (%DS), and late lumen loss (LLL = MLD post-intervention − MLD control). Binary ISR was defined as DS ≥50% in the stented segment.

### Statistical analysis

The sample size calculation was based on the previous TIS study. The 12-month difference in LLL 0.24 (±0.27) mm with alpha type I error of 5%, and beta test strength of 80% was used to determine the required group size of 64 per arm.

Normally distributed continuous variables are presented as mean and standard deviation, and were compared using Student’s two-sample *t*-test. Continuous variables with non-normal distribution are presented as median and range, and were compared using the non-parametric Mann–Whitney U test. Categorical variables are presented as count and percentage, and were compared using the chi-square or Fisher’s exact test. Bonferroni correction was used for multiple comparisons. Kaplan-Meier curves are used to display time-to-event data, which were compared using the log-rank test. Multiple logistic regression (stepwise forward method) was used to identify the most significant predictive factors for repeated binary restenosis, with adjustment for diabetes mellitus and other possible confounding factors. Each odds ratio (OR) is expressed with a 95% confidence interval (CI). A *p* value of <0.05 was considered significant. Evaluation was based on intention-to-treat. All statistical analyses were performed using IBM SPSS Statistics version 22.

## Results

**Fig. 1:**
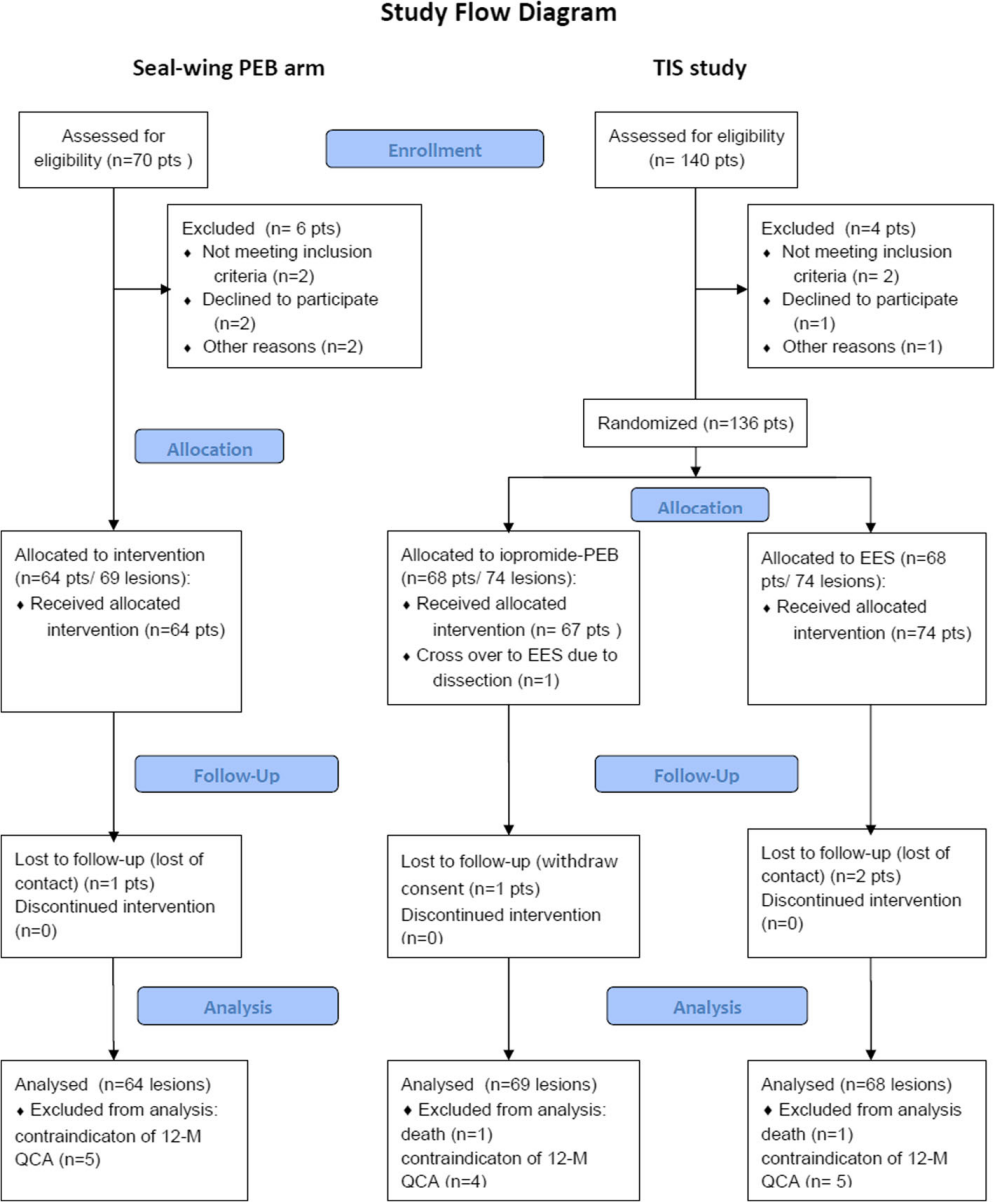
CONSORT study flow diagram

The course of the study is shown in the CONSORT study flow diagram (Fig. 1). We included 64 patients with 69 BMS-ISR lesions, all of whom were treated with seal-wing PEB. Table 1 presents the baseline demographic, clinical, angiographic, and ISR characteristics of the study group, with comparison to the control groups from the TIS study.

**Table 1:** Baseline parameters

	Seal-wing PEB	Iopromide-coated PEB	EES	*p*	
				Seal-wing vs. iopromide- PEB	Seal-wing PEB vs. EES
Demographic parameters					
Patients/ ISR lesions, n	64/69	68/74	68/74		
Male/female	49 (76.56%)/	43 (63.24%)/	46 (67.65%)/	0.288^c^	0.762^c^
	15 (23.44%)	25 (36.74%)	22 (32.35%)		
Age, years	65.25 ± 11.01^a^	65.6 ± 10.9^a^	65.5 ± 10.6^a^	1.000^d^	1.000^d^
Body mass index, kg/m^2^	28.38 ± 4.93^a^/21.19^b^	28.7 ± 4.0^a^/19.23^b^	29.3 ± 4.2^a^/18.61^b^	1.000^f^	0.255^f^
Ejection fraction, %	52.31 ± 10.01^a^/55.00^b^	49.74 ± 11.95^a^/50.0^b^	49.57 ± 11.44^a^/50.0^b^	0.732^f^	0.729^f^
Diabetes mellitus	18 (28.12%)	17 (25.00%)	18 (26.47%)	1.000^c^	1.000^c^
Renal insufficiency	2 (3.12%)	2 (2.94%)	7 (10.29%)	1.000^e^	0.495^e^
CABG	6 (9.38%)	3 (4.41%)	6 (8.82%)	1.000^e^	1.000^e^
Ever smoked	31 (48.44%)	31 (45.59%)	29 (42.65%)	1.000^e^	1.000^c^
Previous MI	31 (48.44%)	43 (63.24%)	41 (60.29%)	0.117^c^	0.258^c^
2VD/3VD	43 (67.19%)	38 (55.88%)	41 (60.29%)	1.000^c^	1.000^c^
Multi ISR	5 (7.81%)	4 (5.88%)	5 (7.35%)	1.000^e^	1.000^c^
Baseline PCI					
ACSy (STEMI/NSTEMI)	37 (57.81%)	45 (66.18%)	50 (73.53%)	0.966^c^	0.171^c^
stable AP	27 (42.19%)	23 (33.82%)	18 (26.47%)		
Type of lesion					
B2/C	47 (68.12%)	51 (68.92%)	47 (63.51%)	1.000^c^	1.000^c^
Lesion localization					
LAD/RD	34 (49.28%)	35 (47.30%)	40 (54.05%)	1.000^e^	1.000^e^
RCx/OM	15 (21.74%)	16 (21.62%)	10 (13.51%)		
RCA	18 (26.09%)	22 (29.73%)	22 (29.73%)		
SVG	2 (2.9%)	1 (1.35%)	2 (2.70%)		
Diameter of the previous stent, mm	3.09 ± 0.48^a^/3.00^b^	3.18 ± 0.43^a^/3.0^b^	3.20 ± 0.41^a^/3.0^b^	0.390^f^	0.225^f^
Length of the previous stent, mm	25.67 ± 15.48^a^/20.00^b^	22.65 ± 11.70^a^/19.0^b^	19.39 ± 9.27^a^/16.0^b^	0.720^f^	0.012^f^
In-stent restenosis					
ACSy, STEMI/NSTEMI	19 (29.69%)	24 (35.29%)	25 (36.76%)	1.000^e^	0.333^c^
Stable AP	42 (65.62%)	41 (60.29%)	33 (48.53%)		
Other, silent ischemia	4 (6.25%)	3 (4.41%)	10 (14.71%)		
Time to ISR, months	12.49 ± 11.06^a^/7.00^b^	12.10 ± 8.47^a^/9.0^b^	16.51 ± 9.49^a^/24.0^b^	1.000^f^	0.042^f^
Type of ISR					
I (focal; all)	25 (36.23%)	30 (40.54%)	21 (28.38%)	1.000^e^	1.000^e^
II (diffuse)	33 (47.83%)	34 (45.95%)	35 (47.30%)		
III (proliferative)	6 (8.7%)	5 (6.76%)	8 (10.81%)		
IV (occlusion)	5 (7.25%)	5 (6.76%)	10 (13.51%)		
Periprocedural parameters					
Cutting predilatation	20 (28.99%)	16 (21.62%)	5 (6.76%)	0.933^c^	0.002^c^
ISR; PEB/EES diameter, mm	3.27 ± 0.47^a^/3.17^b^	3.32 ± 0.39^a^/3.5^b^	3.31 ± 0.43^a^/3.5^b^	1.000^f^	1.000^f^
ISR; PEB/EES length, mm	23.19 ± 12.98^a^/20.00^b^	22.53 ± 8.13^a^/20.0^b^	28.47 ± 12.76^a^/24.0^b^	1.000^f^	0.0003^f^
Postdilatation, atm	13.48 ± 2.34^a^/12.00^b^	14.84 ± 2.77^a^/16.0^b^	14.11 ± 2.45^a^/12.0^b^	0.009^f^	0.411^f^
Second stent implantation	8 (11.59%)	11 (14.86%)	11 (14.86%)	1.000^c^	1.000^c^
Crossover to DES	2 (2.9%)	2 (2.7%)	-	1.000^c^	-

Qualitative data are given as n (%); quantitative data as ^a^mean (± standard deviation) and ^b^median

^c^chi-square test; ^d^Student T-test; ^e^Fisher’s exact test; ^f^Mann–Whitney U test

We obtained 12-month clinical data for 63 patients with BMS-ISR (98.44%; 95%CI: 91.6–99.96%) and 12-month QCA was performed in 64 lesions (92.76%; 95%CI: 83.89–97.61%).

Angiographic parameters are provided in Table 2. Baseline and early post-procedural angiographic results (MLD, RefD, acute gain, and residual%DS) did not significantly differ between the seal-wing PEB and iopromide-coated PEB groups. However, 12-month follow-up results showed significantly lower MLD and RefD, and higher incidence of repeated binary restenosis, higher DS%, and LLL (primary end-point) in the seal-wing PEB group compared to the iopromide-coated PEB group (*p* values: 0.0006, 0.039, 0.012, <0.0001, and < 0.0001, respectively).

**Table 2:** Baseline, postprocedural, and 12-month QCA parameters

		Seal-wing PEB	Iopromide-coated PEB	EES	*p*	
					Seal-wing vs. iopromide- PEB	Seal-wing PEB vs. EES
Patients/lesions, n		59/64	63/69	62/68		
Preprocedural Parameters - ISR						
Minimal lumen diameter, mm	Mean	0.86	0.92	0.79	1.000^a^	0.732^a^
	SD	0.46	0.45	0.48		
	Median	0.93	1.00	0.77		
Reference diameter, mm	Mean	2.50	2.64	2.66	0.240^a^	0.087^a^
	SD	0.43	0.47	0.45		
	Median	2.45	2.63	2.66		
% Diameter stenosis	Mean	74.3	71.8	78.0	0.939^a^	0.372^a^
	SD	14.5	13.9	13.4		
	Median	73.0	70.0	76.0		
Post-procedural Parameters - Post re-PCI						
Minimal lumen diameter, mm	Mean	2.09	2.18	2.51	1.000^a^	<0.0001^a^
	SD	0.45	0.39	0.38		
	Median	1.99	2.13	2.49		
Reference diameter, mm	Mean	2.72	2.79	3.01	0.756^a^	0.0003^a^
	SD	0.42	0.41	0.40		
	Median	2.63	2.79	2.96		
Acute gain, mm	Mean	1.23	1.25	1.72	1^a^	<0.0001^a^
	SD	0.53	0.54	0.47		
	Median	1.19	1.12	1.69		
% Diameter residual stenosis	Mean	21.5	19.5	16.3	0.153^a^	<0.0001^a^
	SD	7.9	7.4	5.9		
	Median	23.5	20.0	16.0		
12-month QCA parameters						
Minimal lumen diameter, mm	Mean	1.63	2.09	2.07	0.0006^a^	0.003^a^
	SD	0.78	0.57	0.80		
	Median	1.68	2.13	2.23		
Reference diameter, mm	Mean	2.62	2.81	2.96	0.039﻿^a^	0.0003^a^
	SD	0.53	0.48	0.50		
	Median	2.56	2.81	2.86		
% Diameter stenosis	Mean	42.4	26.2	30.9	﻿<0.0001^a^	0.009^a^
	SD	27.9	18.0	24.6		
	Median	33.5	22.0	21.5		
Late lumen loss, mm	Mean	0.47	0.09	0.44	<0.0001^a^	1.000
	SD	0.57	0.44	0.73		
	Median	0.30	0.02	0.19		
Binary restenosis (%DS˃50%)	(n/%)	18 (28.12%)	6 (8.7%)	13 (19.12%)	0.012^b^	0.666^b^

^a^Mann–Whitney U test; ^b^chi-square test

With regards to early post-procedure results, compared to the EES group, the seal-wing PEB group showed lower MLD and RefD, and higher residual DS% (*p* values: ≤0.0001, 0.0003, and <0.0001, respectively) due to significantly lower acute gain (*p* ≤ 0.0001). These significant differences in MLD, RefD, and DS% persisted at the 12-month follow-up (*p* values: 0.003, 0.0003, and 0.009, respectively). However, the primary end-point of LLL did not differ between the seal-wing PEB and EES groups (*p* = 1.000). The between-group difference in the incidence of repeated binary restenosis was also non-significant (*p* = 0.666).

Clinical follow-up revealed that compared to the iopromide-coated PEB group, the seal-wing PEB group showed a significantly higher incidence of 12-month MACE (*p* = 0.003) based on the higher incidence of repeated TVR (*p* = 0.009). In contrast, no difference in clinical end-points was found between the seal-wing PEB and EES groups (*p* = 0.102) (Table 3).

**Table 3:** 12-month clinical follow-up

		Seal-wing PEB	Iopromide-coated PEB	EES	*p*	
					Seal-wing vs. iopromide- PEB	Seal-wing PEB vs. EES
Patients/lesions, n		63/64	68/74	68/74		
MACE all, n(%)		17 (26.98%)	7 (10.29%)	13 (19.12%)	0.003^a^	0.102^a^
CV death, n(%)		0	1 (1.47%)	1 (1.47%)	1.000^b^	1.000^b^
AIM, n(%)		4 (6.35%)	1 (1.47%)	1 (1.47%)	0.468^b^	0.468^b^
TVR, n(%)		13 (20.63%)	5 (7.35%)	11 (16.18%)	0.009^a^	0.360^a^
Event-free survivors, n(%)		46 (73.02%)	61 (89.71%)	55 (80.88%)	0.027^a^	0.666^a^
AP (CCS), n(%)	0–1	38 (82.61%)	48 (78.69%)	43 (78.18%)	1.000^b^	1.000^b^
	2	7 (15.22%)	13 (21.31%)	12 (21.82%)		
NYHA, n(%)	1	13 (28.26%)	14 (22.95%)	20 (36.36%)	1.000^b^	1.000^b^
	2	31 (67.39%)	44 (72.13%)	31 (56.36%)		
	3	2 (4.35%)	3 (4.92%)	4 (7.27%)		

^a^chi-square test; ^b^Fisher’s exact test

**Fig. 2:**
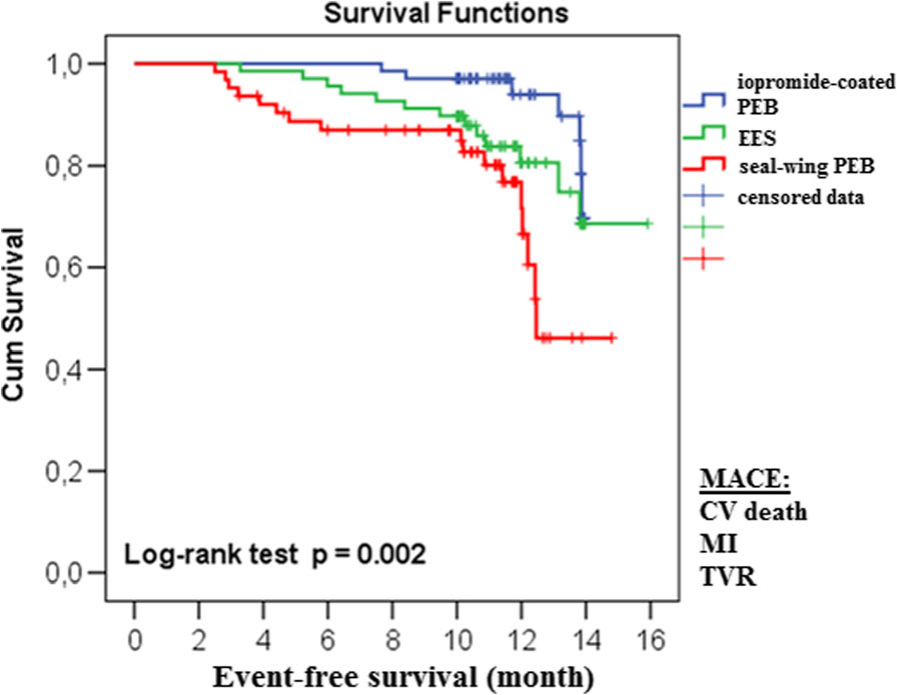
Event-free survival

Figure 2 presents estimates of event-free survival (EFS) among patients with BMS-ISR. The log-rank test revealed significant differences between treatment groups in terms of EFS (time to MACE, *p* = 0.002). This difference was mainly based on the significantly lower average EFS in the seal-wing PEB group (13.67 months; 95%CI: 13.39–13.94) compared to the iopromide-coated PEB group (12.17 months; 95%CI: 11.16–13.19; *p* = 0.0007). There was no difference between the seal-wing PEB and EES groups (14.22 months; 95% CI 13.40–15.05; *p* = 0.218).

The 12-month LLL was significantly higher in the seal-wing PEB group compared to the iopromide-coated PEB group, even in the high-risk subgroups: diabetes patients, ISR lesions ˃10 mm (type II–IV), and vessel diameter < 3 mm (*p* values: 0.024, 0.0006, and <0.0001, respectively). On the other hand, no difference in LLL was found between the seal-wing PEB and EES high-risk subgroups (Table 4).

**Table 4:** Subgroup analysis of 12-month LLL

		Seal-wing PEB	Iopromide-coated PEB	EES	Seal-wing vs. iopromide- PEB	Seal-wing PEB vs. EES
Diabetes mellitus					p^a^	
Patients/lesions, *n*		18/19	16/16	15/16	0﻿.024	0.450
Late lumen loss, mm	Mean	0.64	0.12	0.48		
	SD	0.63	0.33	0.86		
	Median	0.33	0.06	0.12		
ISR length >10 mm						
Patients/lesions, *n*		36/41	42/44	44/47	0.0006	1.000
Late lumen loss, mm	Mean	0.53	0.16	0.53		
	SD	0.57	0.50	0.67		
	Median	0.37	0.05	0.26		
Vessel diameter < 3 mm						
Patients/lesions, *n*		49/53	49/54	47/52	﻿<0.0001	0.564
Late lumen loss, mm	Mean	0.53	0.12	0.42		
	SD	0.60	0.48	0.63		
	Median	**0.37**	0.05	0.16		

^a^Mann–Whitney U test

Logistic regression analysis was performed to assess the impact of various risk factors on the incidence of repeated binary restenosis after seal-wing PEB treatment of ISR, with correlations to each individual parameter (Table 5). Diabetes mellitus was found to be important predictor of repeated binary restenosis (unadjusted OR: 3.018; 95%CI: 1.117–8.156; *p* = 0.029). With adjustment for other confounding risk factors, patients with lesion length > 10 mm had significantly higher chances of repeated binary restenosis occurence (adjusted OR: 3.375; 95%CI: 1.011–11.270; *p* = 0.048).

**Table 5:** Logistic (separately for each parametr) and multivariate logistic regression analysis (the stepwise forward method) for the seal-wing PEB group

Logistic regression analysis			
	*p*	Unadjusted OR	95% CI
Diabetes mellitus (1 = yes, 0 = no)	0.029	3.018	1.117–8.156
Type B2/C lesion (1 = yes, 0 = no)	0.172	2.200	0.709–6.825
Vessel diameter < 3 mm (1 = yes,0 = no)	0.611	1.354	0.422–4.348
ISR length > 10 mm (1 = yes, 0 = no)	0.245	1.778	0.673–4.694
Multivariate logistic regression analysis			
	*p*	adjusted OR	95% CI
ISR length > 10 mm (1 = yes, 0 = no)	0.048	3.375	1.011–11.270

## Discussion

Paclitaxel is an effective antiproliferative agent in cases of DEB. This drug is highly lipophilic and rapidly penetrates into the tissues, with the utilized concentrations stabilizing at 3 μg/mm [12]. The main factor influencing the PEB efficacy is the method used to bind paclitaxel to the balloon catheter surface. In the original concept described by Scheller et al., paclitaxel was bound via the hydrophilic contrast agent iopromide (Paccocath®), which increased its solubility and vascular wall penetration [12].

Preclinical studies show a significantly lower LLL, and reduced neointima maximum thickness and area (*p* values: 0.001, 0.002, and ˂0.001, respectively), with iopromide-coated PEB (Paccocath®) compared to with uncoated PEB (DIOR®) [3].

The efficacy of BMS-ISR treatment using iopromide-coated PEB has been demonstrated in comparison with POBA or paclitaxel-eluting stents (PES) [13, 14].

In the RIBS V trial, patients with BMS-ISR were treated with iopromide-coated PEB or EES. The patients treated with EES showed significantly higher 9-month MLD (*p* < 0.001) and lower %DS (*p* < 0.001). However, these treatment groups did not significantly differ in LLL (*P* = 0.14), incidence of binary restenosis (*p* = 0.22),12-month MACE (*p* = 0.6), or TVR (*p* = 0.22) [15].

Contrary to the RIBS V, in our TIS study the use of iopromide-coated PEB for treatment for BMS-ISR was associated with significantly reduced 12-month LLL compared to implantation of second-generation EES (*p* = 0.0004). However, between-group differences in the incidence of repeated binary restenosis (*p* = 0.078) and 12-month MACE (*p* = 0.213) were non-significant [3].

Several studies comparing PEBs with different coating were published. Sub-analysis of the SCAAR registry revealed that iopromide-coated PEB (SeQuent*®*Please; paclitaxel 3 μg/mm^2^) for treatment of ISR lesions was associated with a lower risk of binary restenosis (adjusted HR: 0.48; 95%CI: 0.23–0.98) compared to uncoated PEB (Elutax©; paclitaxel 2 μg/mm^2^) [16].

Nijhoff et al. found that the treatment of BMS/DES-ISR using urea-coated PEB were associated with a significantly lower 6-month LLL (*p* = 0.014), higher value of FFR distally (*p* = 0.029), and a lower percentage volume of intimal hyperplasia (*p* = 0.006) compared to shellac-coated PEB. No difference in the incidence of repeated binary restenosis (*p* = 0.16) and only a trend towards lower TLR were observed (*p* = 0.057) [17].

Our results confirm that iopromide coating influenced the efficacy of PEB in ISR treatment. We demonstrated that patients with BMS-ISR showed significantly higher 12-month LLL, incidence of repeated binary restenosis, 12-month MACE and TVR following treatment with seal-wing PEB compared to iopromide-coated PEB. This unfavorable effect on reducing LLL was also found in high-risk subgroups of patients with diabetes, ISR length >10 mm, and artery diameter < 3 mm.

Within the seal-wing PEB group, post-dilation pressures were significantly lower than those used in the iopromide-coated PEB group (*p* = 0.009); however, it does not seem to play a mayor role because the early post-procedural angiographic results (MLD, acute gain, and %DS) did not differ between the groups.

Based on the obviously significant inferior angiographic results, we cannot recommend the use of seal-wing PEB instead of iopromide-coated PEB in the treatment of BMS-ISR.

The main reason of different resuls in our PEB arms may be the method of paclitaxel binding to the balloons. The seal-wing technology of paclitaxel aplication directly on the rough balloon sufrace before folding with only hydrophilic lubrication seems not to be as effective as paclitaxel-iopromide coating.

Compared to EES, seal-wing PEB treatment was associated with a significantly lower 12-month MLD, presumably due to poorer early post-procedural results (post-procedural MLD and acute gain), but the groups did not significantly differ in the primary end-point of 12-month LLL.

In the recently published PRODIGY study, long-term (24 month) dual antiplatelet therapy (DAPT) after ISR treatment was associated with a significantly lower composite end-point (death, non-fatal MI or cerebrovascular accident: 16.7% vs. 7.3%; *p* = 0.034) compared to short-term (6 month) DAPT [18, 19]. In our study, duration of DAPT differed according the the treatment regimen (3 month after PEB and 12 month after EES implantation) and it was temporarily interrupted in 2 patients (3,1%) in the seal-wing PEB arm one month after PCI due to minor bleeding and tooth extraction.

The differences in the incidence of 12-month MACE between seal-wing PEB and EES groups did not reach statistical significance, however.

Subanalysis of the PRODIGY study comparing the first and second generation of DES in the treatment of ISR, found a significantly lower 24 month target lesion failure (TLF; death, target vessel myocardial infarction or clinically driven target lesion revascularization) after EES (Xience; Co/Cr metallic platform) compared to PES or ZES implantation (8% vs. 20% and 26%; EES vs. PES: *p* = 0.05 and EES vs. ZES: *p* = 0.001, respectively), principally due to the lower occurrence of clinically driven TLR in EES group (5% vs. 13% and 20%; EES vs. PES: *p* = 0.09 and EES vs. ZES: *p* = 0.03, respectively) [20–22]. We achieved higher rate of MACE (19.12%) and also TVR (16.18%) in EES-treated BMS-ISR group, even in shorter follow-up period (12 month). A possible influence of different metallic platform (Pt/Cr alloy) of used EES should need further investigation.

### Limitations

Our study has several limitations. In particularly, it was a non-randomized study that compared active treatment groups with control arms from the previous TIS study. Nevertheless, selection bias likely did not play a major role, since the patient cohorts (particularly the seal-wing PEB and iopromide-coated PEB groups), did not differ with respect to main baseline parameters.

Diferences in clinical outcomes between the study arms should be taken with caution, as our study was not powered for clinical endpoints. The results of the logistic regression analysis in different subgroups should also be interpreted with caution.

## Conclusions

Treatment of BMS restenosis using seal-wing PEB led to significantly higher 12-month LLL, repeated binary restenosis, 12-month TVR, and MACE compared to treatment with iopromide-coated PEB. However, no significant differences were found in comparison with EES.
